# Global trends and hotspots in research on acupuncture for neurodegenerative diseases over the last decade: a bibliometric and visualization analysis

**DOI:** 10.3389/fnagi.2024.1390384

**Published:** 2024-05-10

**Authors:** Qian Tan, Xinyuan Liu, Shuyu Xu, Jiangmin Chen, Weixian Li, Shuai Zhang, Yanjun Du

**Affiliations:** ^1^College of Acupuncture and Orthopedics, Hubei University of Chinese Medicine, Wuhan, China; ^2^Hubei Shizhen Laboratory, Wuhan, China

**Keywords:** acupuncture, neurodegenerative diseases, bibliometric analysis, CiteSpace, VOSviewer, visual analysis

## Abstract

**Objectives:**

This study aimed to explore the current status and trends of acupuncture for neurodegenerative diseases (NDs) in the last decade and provide new insights for researchers in future studies.

**Methods:**

The publications concerning acupuncture treatment for NDs published between 2014 and 2023 were extracted from the Web of Science Core Collection. We used CiteSpace and VOSviewer to analyze data on numbers of annual publications, countries, institutions, cited journals, cited authors, cited references, keywords, and citation bursts about acupuncture for NDs.

**Results:**

A total of 635 publications were obtained from 2014 to 2023. We identified the most prolific journals, countries, institutions, authors, patterns of authorship, and the main direction of future research in the field of acupuncture for NDs in the last decade. The country, institution, and journal with the most publications are China (389 articles), Beijing University of Chinese Medicine (56 articles), and Evidence Based Complementary and Alternative Medicine (42 articles), respectively. The high-frequency keywords focused on “Alzheimer's disease,” “Parkinson's disease,” “acupuncture,” “dementia,” and “electroacupuncture.” The top five keywords in terms of centrality were “cerebral ischemia,” “acupuncture stimulation,” “fMRI,” “apoptosis,” and “deep brain stimulation.”

**Conclusion:**

The results from this bibliometric study provide insight into the research trends in acupuncture therapy for NDs, and the current status and trends of the past decade, which may help researchers confirm the current status, hotspots, and frontier trends in this field.

## Introduction

Neurodegenerative diseases (NDs) are a heterogeneous group of neurological disorders (Wilson et al., 2023), including Alzheimer's disease (AD) (Knopman et al., 2021), Parkinson's disease (PD) (Poewe et al., 2017), Huntington's disease (HD) (Bates et al., 2015), and amyotrophic lateral sclerosis (ALS) (Kiernan et al., 2011). AD is considered the most frequent type of ND, occurring in 60% to 80% of all cases. These diseases are diverse in their pathophysiology (Gitler et al., 2017), but all of them entail the progressive loss of neurons in the central nervous system (CNS) or peripheral nervous system (PNS) (Wilson et al., 2023). Neuronal loss and progressive degeneration of different areas of the nervous system result in the breakdown of the core communicative circuitry, culminating in impaired memory, cognition, behavior, sensory, and/or motoric function. NDs are a major cause of death and disability worldwide, influenced by many factors including age, genetics, and injuries. The adverse effects of NDs affect the lives of millions of people worldwide. In particular, in the elderly population, NDs are common (Hou et al., 2019). The prevalence of NDs is expected to rise with the increasing life expectancy in most countries. Approximately more than 50 million people are currently affected by NDs (Nichols et al., 2019), which will almost triple to 152 million in 2050 if no effective preventive or therapeutic solutions are found (Livingston et al., 2020). NDs are considered separate clinical entities that target different brain regions with distinct pathology and symptoms (Bordoni et al., 2020); based on the genetic, molecular, or cellular level, some studies found that certain players may influence disease onset and progression by interacting with the major pathological hallmarks of NDs, such as blood–brain barrier breakdown (Sweeney et al., 2018), aggregation and spread of misfolded proteins (Vaquer-Alicea and Diamond, 2019), selective vulnerability of particular neurons (Chi et al., 2018), and activation of immune responses (Tang and Le, 2016).

Despite a lot of the preclinical studies and clinical attempts, the identification of effective therapeutics has not yet emerged, which might have resulted from the complex multifactorial nature of NDs (Sengupta and Kayed, 2022). The currently available treatments are primarily treatments of symptoms, rather than actual curative therapies (Passeri et al., 2022). However, some medicines may not have significant effects on the advanced disease stage of patients over time. Therefore, it is a challenge to find an effective alternative therapy for ameliorating symptoms of NDs. Acupuncture, as one of the most popular treatments applied in traditional Chinese medicine, has been applied clinically for over a millennium. By stimulating specific parts of the body, acupuncture could inhibit the inflammatory reaction (Jung et al., 2021), reduce oxidative stress injury (Du et al., 2018), restrain apoptosis (Sun et al., 2020), promote neural and vascular regeneration (Jang et al., 2020b), and so on. There are many stimulation methods of acupoint, such as handle acupuncture, electroacupuncture, warm acupuncture, and so on. Recently, acupuncture has been regarded as an alternative therapy with small side effects and obvious curative effects. Its therapeutic effects on NDs have been validated by both basic (Jang et al., 2020b; Zheng et al., 2021) and clinical studies (Jia et al., 2017). In evidence-based medicine research (Lu et al., 2022), acupuncture has many indications, including NDs caused by various reasons. Nevertheless, little attention has been paid to topic hotspots and trends in acupuncture for NDs.

Bibliometrics is a statistical analysis and quantitative tool to study publications through mathematical and statistical methods for tracking the development of a certain research field over a defined time frame (Yang et al., 2023). It laid special stress on analyzing relevant information of this research field, for example, the impacts of publications, contributions of authors/institutes/countries, patterns of authorship, and the main direction of future research in the field (Zhang et al., 2020). Based on the Web of Science Database, the cross-science of quantitative analysis can show the global research trends and topic hotspots of a research field with analysis software. CiteSpace and VOSviewer, as visual analysis software, are widely used in bibliometric analysis, which was developed by Chen and Chen (2005) and van Eck and Waltman (2010), respectively.

In the present study, we conducted a bibliometric analysis of studies on the use of acupuncture treatment for NDs over the past decade at a global level to illustrate the research landscape and explore the hot topics and emerging trends.

## Methods

### Data acquisition

All publications were obtained from the Web of Science Core Collection including SCI-EXPANDED, SSCI, AandHCI, CPCI-S, CPCI-SSH, BKCI-S, BKCI-SSH, ESCI, and CCR-EXPANDE. The data search strategy included the topic “neurodegenerative diseases” and “acupuncture therapy,” and all extracted studies were published in the last decade. Here were the search strategies: 1# TS = ((Acupunctur^*^) OR (Acupunctur^*^ Treatment^*^) OR (Acupunctur^*^ Therap^*^) OR (body Acupunctur^*^) OR (Needle^*^ Acupunctur^*^) OR (Manual^*^ Acupunctur^*^) OR (Acupunctur^*^ Point^*^) OR (Electroacupunctur^*^) OR (Warm^*^ Acupunctur^*^) OR (electr^*^-acupunctur^*^)), 2# TS = ((Neurodegenerative^*^disease^*^) OR (neurodegenerat^*^) OR (Alzheimer^*^ disease^*^) OR (Parkinson^*^ disease^*^) OR (multiple sclerosis^*^) OR (Huntington^*^ disease^*^) OR (amyotrophic lateral sclerosis^*^)), timespan = 2014–2023, 1# AND 2#, language was English.

### Analysis method

CiteSpace (version 6.2 R2 64-bit) and VOSviewer (version 1.6.18 64-bit) were used for bibliometric analysis and network visualization. The resulting data were imported into Microsoft Excel 2019 for graph generation.

The parameters of CiteSpace were as follows: Time-slicing was performed from January 2014 to December 2023 (1 year per slice), all options in the term source were selected, one node type was selected at a time, selection criteria (top 50 objects). Nodes and links were used to generate visualization maps. The node size represented the publications of authors, institutions, or countries/regions, and the links between them denoted the cooperation relationship in the analysis of countries/regions, institutions, and authors. In the co-citation analysis (including the analysis of cited journals, cited authors, and references), the size of nodes reflected the number of citations in articles, authors, or journals. The connection links between nodes were regarded as the co-occurrence or co-citation relationship. Nodes with high betweenness centrality (>0.1) were usually considered turning points or pivotal points in a field. When the CiteSpace default number of network nodes was >350, the centrality calculation function would be closed. We need to manually click the “compute node centrality” function in the node menu.

VOSviewer is a software program that functions similarly to Citescpace; it could work together with the visual maps created by CiteSpace and reflects collaborative and scholarly relationships.

## Results

### Numbers of annual publications

**Figure 1:**
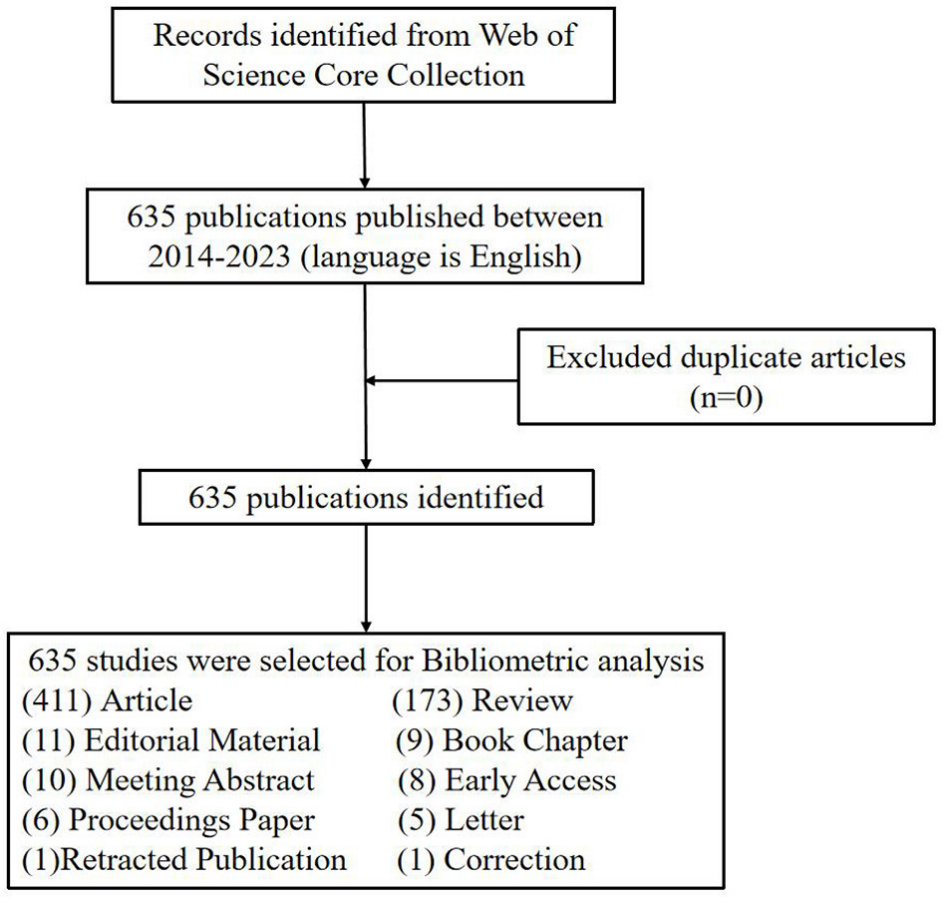
Flowchart of the selection process for the eligible publications.

**Figure 2:**
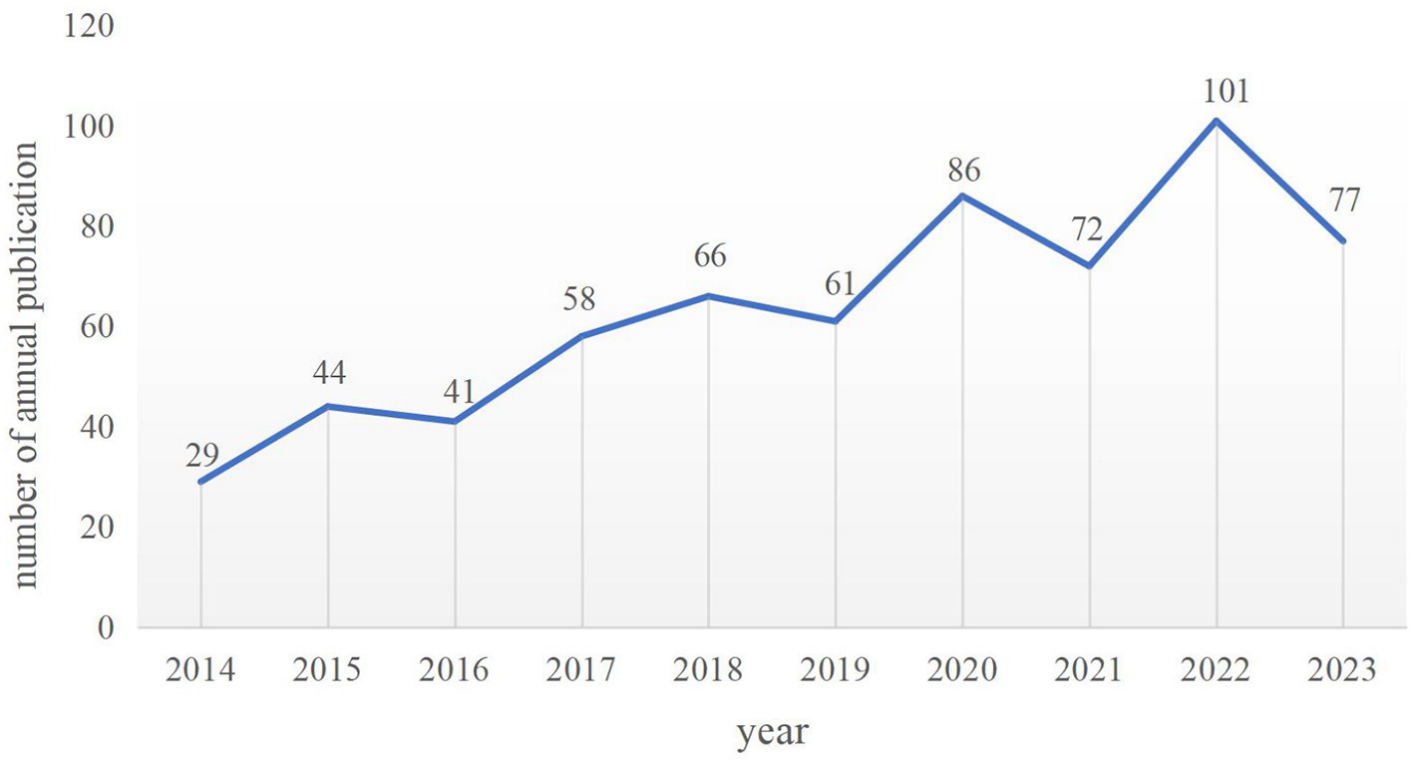
Annual number of publications on acupuncture for NDs from 2014 to 2023. Analysis of Countries/regions.

By searching the database, a total of 635 articles matching this study were included and the type of publications was listed (Figure 1). The number of published works of each year is shown in Figure 2. As can be seen from the figure, the fluctuation of the overall trend was growth. Although the number of publications in some years had declined including 2016, 2019, and 2021, compared with their previous year. The highest number of articles will be issued in 2022, reaching 101. The result showed that acupuncture treatment has received increasing attention in recent years, and the efficacy of acupuncture for the treatment of NDs has also been the subject of more research.

**Figure 3:**
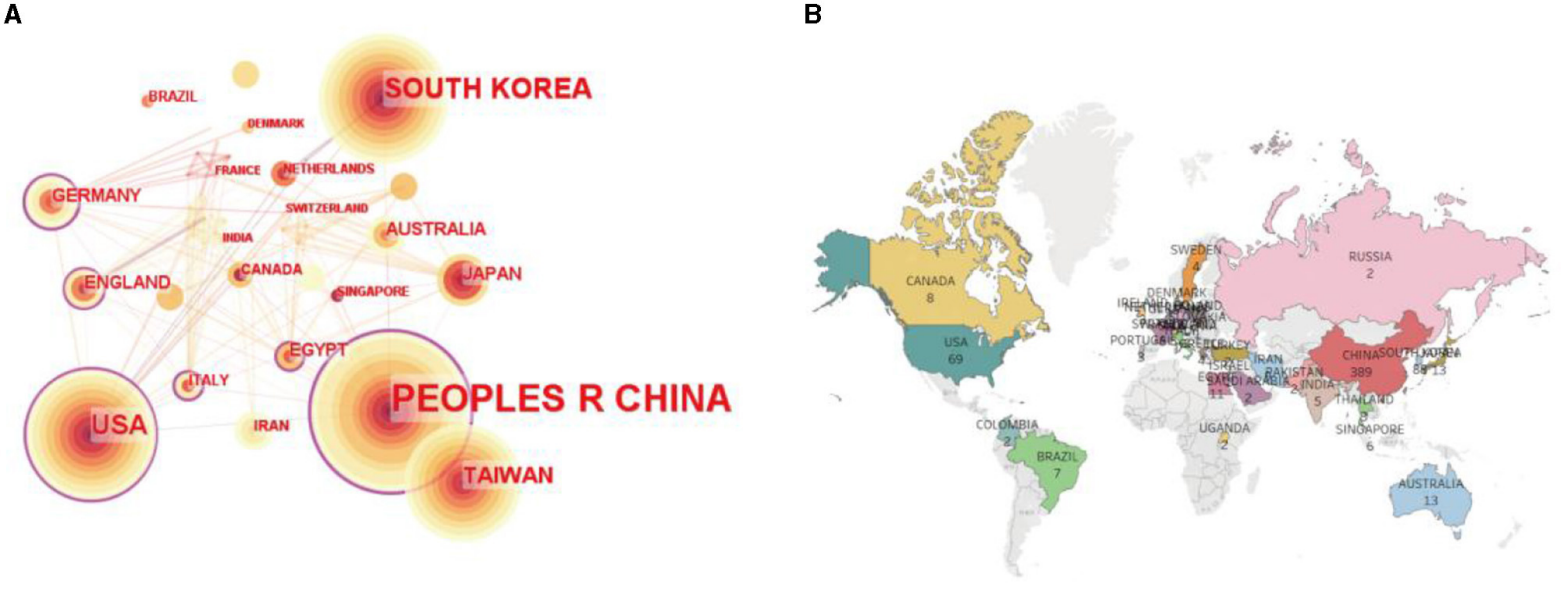
**(A)** Map of countries/regions researching acupuncture for NDs from 2014 to 2023 by CiteSpace. The purple ring represented centrality, which was considered a pivotal point. **(B)** World map of publications distributed in various countries/regions researching acupuncture for NDs from 2014 to 2023.

The 635 references were published by 51 countries/regions. A countries/regions distribution map was generated with CiteSpace, including 51 nodes and 171 links (Figure 3A). A detailed overview was presented as a world map used by the Tableau Public (Figure 3B). Table 1 shows the top 10 most prolific and centrality countries/regions in the field related to acupuncture for NDs. Among them, most publications come from China (*n* = 389), where acupuncture originated. In South Korea, the USA, Germany, and Australia, researchers also pay more attention to acupuncture as a specific treatment for NDs (Table 1). The top five countries/regions for centrality were China (0.57), Germany (0.22), Italy (0.20), England (0.19), and USA (0.18).

**Table 1:** Top 10 most prolific and the highest centrality of countries/regions in the field related to acupuncture for NDs.

**Rank**	**Publication**	**Countries/regions**	**Rank**	**Centrality**	**Countries/regions**
1	389	Peoples R China	1	0.57	Peoples R China
2	88	South Korea	2	0.22	Germany
3	69	USA	3	0.20	Italy
4	31	Taiwan	4	0.19	England
5	16	Germany	5	0.18	USA
6	13	Australia	6	0.18	Egypt
7	13	Japan	7	0.07	Taiwan
8	12	England	8	0.07	Greece
9	11	Egypt	9	0.07	Malaysia
10	9	Iran	10	0.06	Australia

### Analysis of institutions

**Figure 4:**
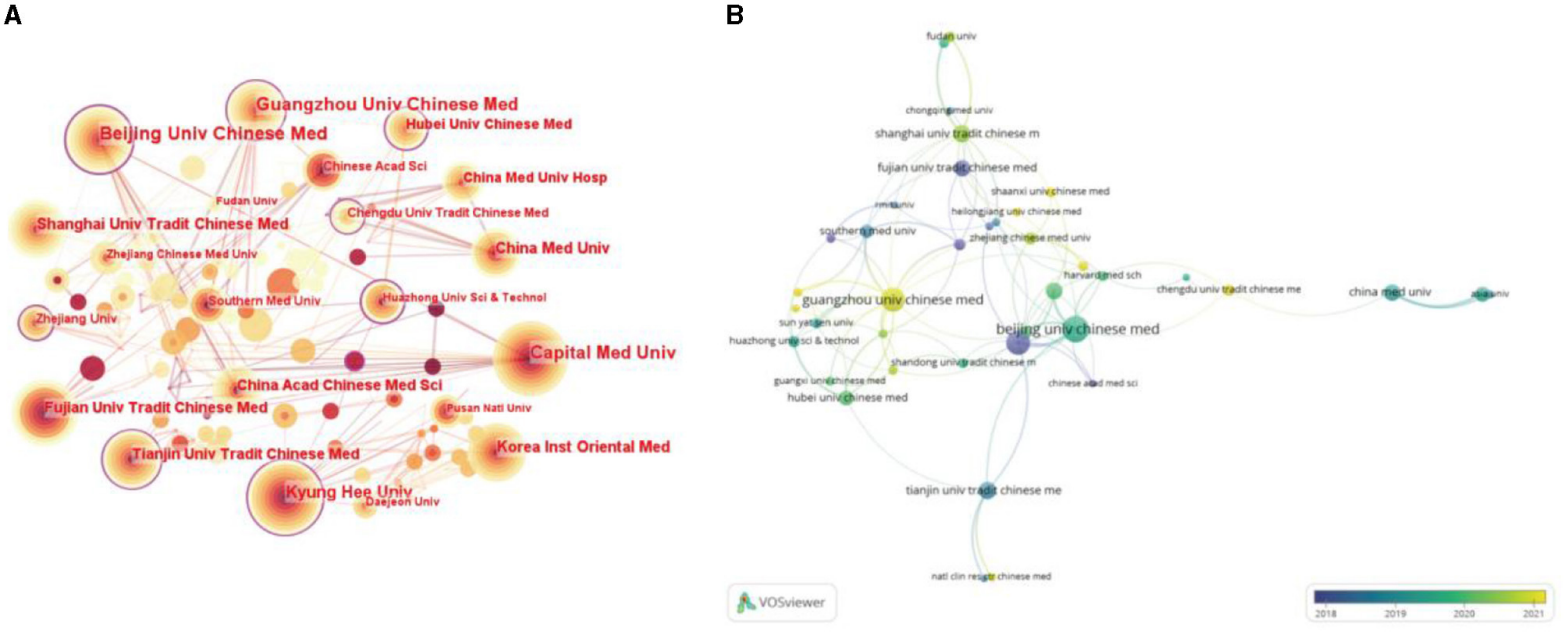
**(A)** Map of institutions researching acupuncture for NDs from 2014 to 2023 by CiteSpace. The purple ring represented centrality, which was considered a pivotal point. **(B)** Overlay visualization of institutions researching acupuncture for NDs from 2014 to 2023 by VOSviewer. The degree of node dispersion reflects the cooperation degree between institutions.

The distribution map of the institutions consisted of 289 nodes and 451 links, which indicated that 289 institutions participated in research studies on acupuncture for NDs (Figure 4A). The cooperation between institutions was not very close, especially the lack of cooperation between institutions from different countries (Figure 4B). The top five most prolific institutions were the Beijing University of Chinese Medicine, the Guangzhou University of Chinese Medicine, the Capital Medical University, the Kyung Hee University, and the Korea Institute of Oriental Medicine. Among them, the Beijing University of Chinese Medicine had the largest number of publications in this field (*n* = 56). Meanwhile, the top five institutions in terms of centrality were the Beijing University of Chinese Medicine (0.22), the Huazhong University of Science and Technology (0.22), the Harvard Medical School (0.21), the Kyung Hee University (0.20), and the Guangzhou University of Chinese Medicine (0.19). In particular, the Beijing University of Chinese Medicine was the institution with the highest centrality and highest number of publications, indicating that it is an important institution in the research on acupuncture for NDs (Tables 2, 3).

**Table 2:** Top 10 most prolific institutions in the field related to acupuncture for NDs.

**Rank**	**Publications**	**Institutions**
1	56	Beijing University of Chinese Medicine
2	49	Guangzhou University of Chinese Medicine
3	47	Capital Medical University
4	40	Kyung Hee University
5	25	Korea Institute of Oriental Medicine
6	25	Shanghai University of Tradition Chinese Medicine
7	24	China Medical University
8	23	Fujian University of Tradition Chinese Medicine
9	23	China Academy Chinese Medical Sciences
10	22	Tianjin University of Tradition Chinese Medicine

**Table 3:** Top 10 highest centrality of institutions in the field related to acupuncture for NDs.

**Rank**	**Centrality**	**Institutions**
1	0.22	Beijing University of Chinese Medicine
2	0.22	Huazhong University of Science and Technology
3	0.21	Harvard Medical School
4	0.20	Kyung Hee University
5	0.19	Guangzhou University of Chinese Medicine
6	0.16	Chengdu University of Tradition Chinese Medicine
7	0.13	Tianjin University of Tradition Chinese Medicine
8	0.11	Hubei University of Chinese Medicine
9	0.11	Zhejiang University
10	0.09	Capital Medical University

### Analysis of journals and cited journals

**Figure 5:**
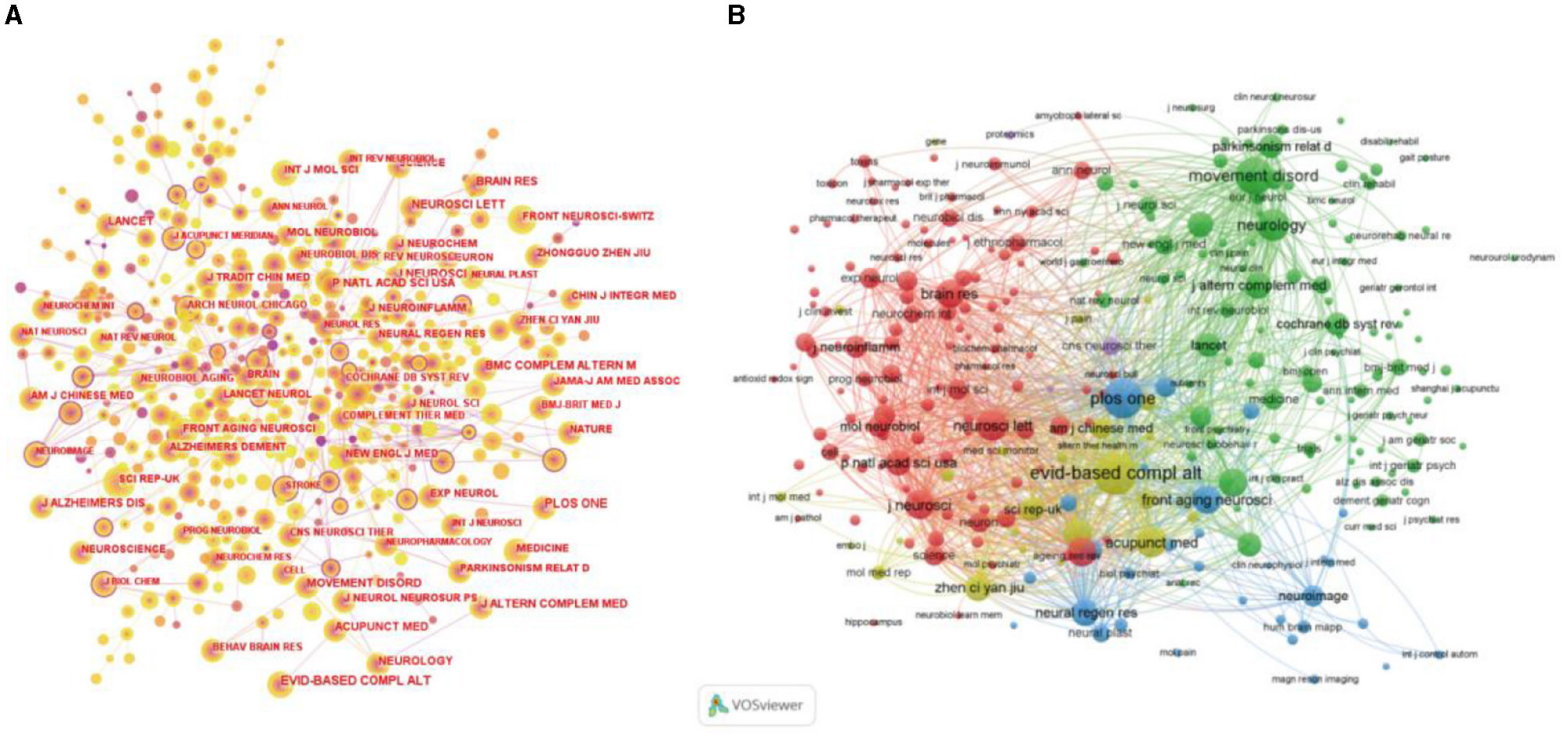
**(A)** Cited journal maps related to acupuncture for NDs from 2014 to 2023 by CiteSpace. The purple ring represents centrality, which is considered a pivotal point. **(B)** Network visualization of Cited journals related to acupuncture for NDs from 2014 to 2023 by VOSviewer. The link indicates the co-citation relationship between cited journals, and the same color of node represents the same cluster.

The top 10 journals on acupuncture for NDs are listed in Table 4. Evidence-Based Complementary and Alternative Medicine was the most prolific journal, with 42 articles, followed by Medicine with 35 articles. A Cited Journals map was generated via CiteSpace and VOSviewer (Figure 5). The top five cited journals with the highest citations and highest centrality of cited journals are shown in Table 5. The top-ranked cited journals for frequency and centrality were PLoS One (*n* = 352) and Human Brain Mapping (0.31), respectively.

**Table 4:** Top 10 scholarly journals related to acupuncture for NDs.

**Rank**	**Journal**	**Publications**	**IF (2023)**
1	Evidence-based complementary and alternative medicine	42	NA
2	Medicine	35	1.6
3	Frontiers in aging neuroscience	32	4.8
4	Acupuncture in medicine	21	2.5
5	Frontiers in neurology	19	3.4
6	Frontiers in neuroscience	14	4.3
7	Neural plasticity	12	3.1
8	Neural regeneration research	12	6.1
9	Chinese journal of integrative medicine	11	2.9
10	BMC complementary and alternative medicine	10	NA

**Table 5:** Top 10 cited journals with the highest citations and data on co-citation centrality related to acupuncture for NDs.

**Rank**	**Frequency**	**Journal**	**Rank**	**Centrality**	**Journal**
1	352	Plos One	1	0.31	Human Brain Mapping
2	328	Evidence-based Complementary and Alternative Medicine	2	0.26	Journal of Physiological Sciences
3	236	Neurology	3	0.25	Journal of Internal Medicine
4	214	BMC Complementary and Alternative Medicine	4	0.19	Physiology and Behavior
5	211	Brain Research	5	0.19	American Journal of Psychiatry
6	208	Acupuncture in Medicine	6	0.18	Journal of Clinical Investigation
7	203	Neuroscience Letters	7	0.17	Neuroimage
8	187	Journal of Alternative And Complementary Medicine	8	0.17	Clinical Neurology and Neurosurgery
9	177	Journal of Neuroscience	9	0.16	Clinical Rehabilitation
10	174	Lancet	10	0.16	Frontiers in Cellular Neuroscience

### Analysis of authors

**Figure 6:**
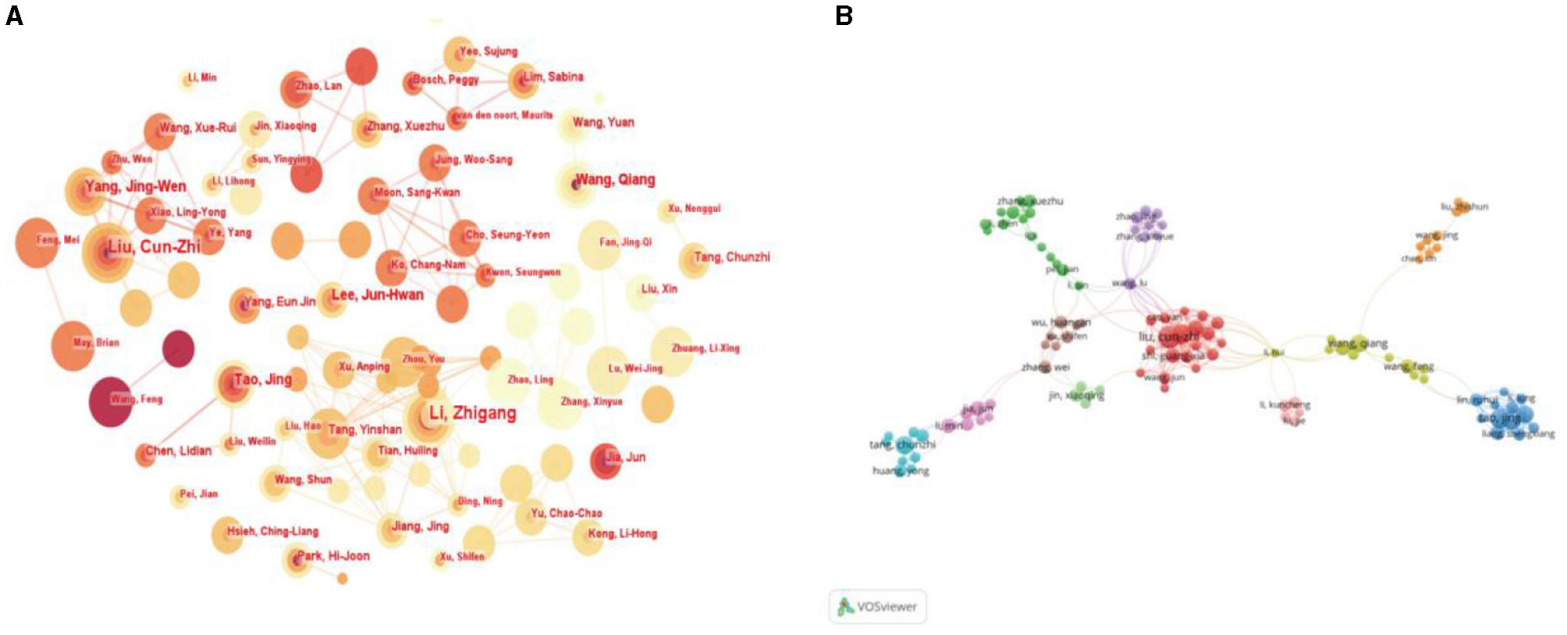
**(A)** Map of authors related to research acupuncture for NDs from 2014 to 2023 by CiteSpace. **(B)** Network visualization of authors related to acupuncture for NDs from 2014 to 2023 by VOSviewer. The link indicates the collaborated relationship between the authors, and the same color of the node represents the same cluster.

In total, 371 authors were involved in studies on acupuncture for NDs. The author map is shown in Figure 6. The node size represents the publications of authors. The links of the figures provided information about influential research groups and potential collaborators, and the collaborations among authors. From Figure 6, it can be seen that active and close cooperation was common among authors in this field. The top 10 most prolific authors in the field are shown in Table 6. Among them, the top five authors in terms of the number of publications were Li Zhigang, Liu Cunzhi, Yang Jingwen, Wang Qiang, and Tao Jing. The most prolific author was Li Zhigang from the Beijing University of Chinese Medicine, having published 18 articles in this field.

**Table 6:** Top 10 most prolific authors in the field related to acupuncture for NDs.

**Rank**	**Publications**	**Authors**	**Affiliation of the authors**
1	18	Li, Zhigang	Beijing University of Chinese Medicine
2	15	Liu, Cun-Zhi	Beijing University of Chinese Medicine
3	11	Yang, Jing-Wen	Beijing University of Chinese Medicine
4	9	Wang, Qiang	The Fourth Military Medical University
5	8	Tao, Jing	Fujian University of Traditional Chinese Medicine
6	8	Lee, Jun-Hwan	Korea Institute of Oriental Medicine
7	7	Chen, Lidian	Fujian University of Traditional Chinese Medicine
8	7	Jiang, Jing	Beijing University of Chinese Medicine
9	7	Park, Hi-Joon	Kyung Hee University
10	6	Zhang, Xuezhu	Tianjin University of Traditional Chinese Medicine

### Analysis of cited authors

**Figure 7:**
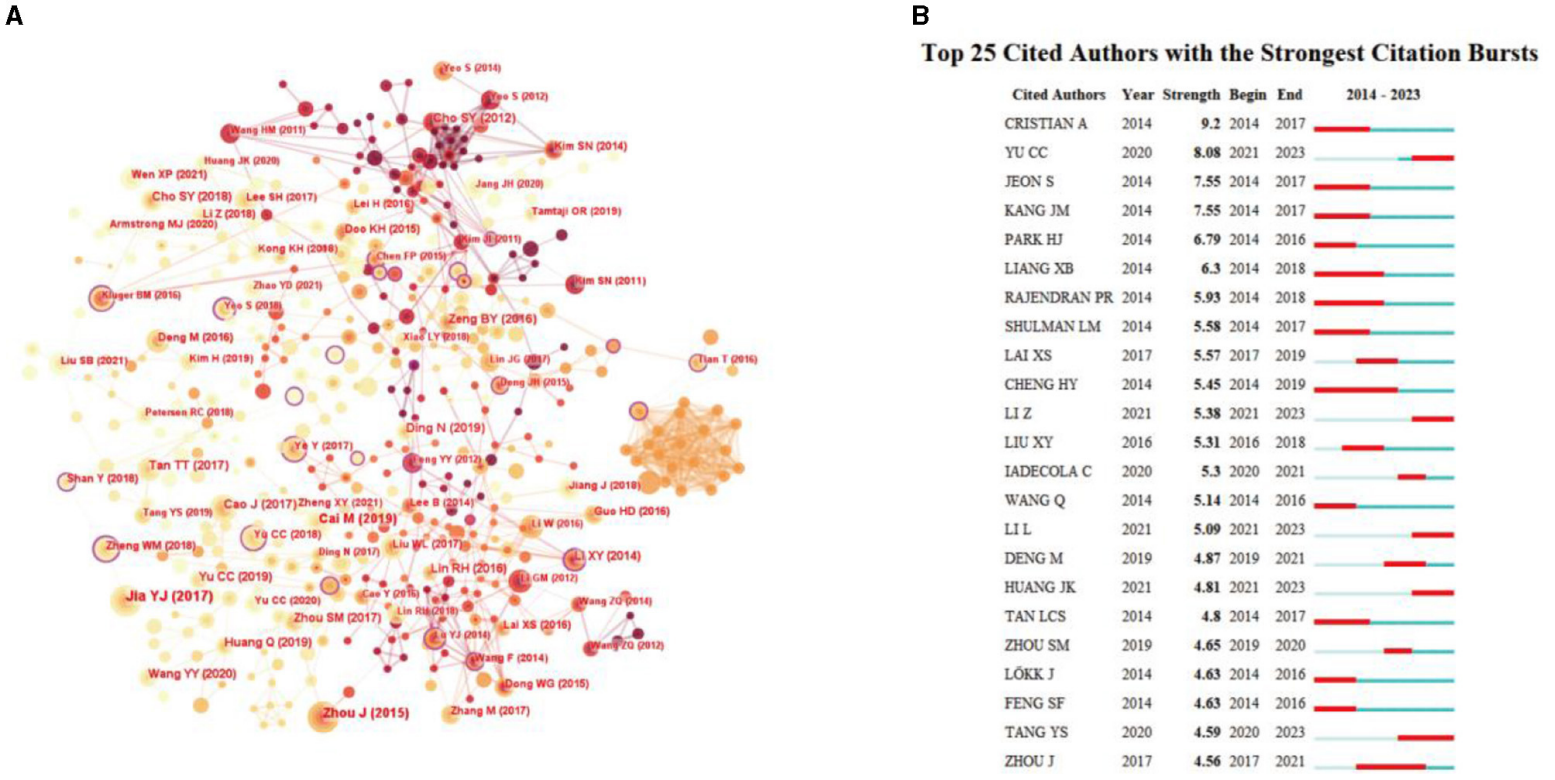
**(A)** Map of cited authors related to research acupuncture for NDs from 2014 to 2023 by CiteSpace. **(B)** Strongest citation bursts of the top 25 cited authors related to research acupuncture for NDs from 2014 to 2023 by CiteSpace.

We used CiteSpace software to generate a cited author map consisting of 493 nodes and 2908 links (Figure 7). The top five cited authors with the highest citations were Seung-Yeon Cho, Myeong Soo Lee, Jing Zhou, Wang Ying, and Bai-Yun Zeng (Table 7). The top five cited authors ranked according to centrality were Christopher G Goetz (0.33), Jong-In Kim (0.33), Xuying Li (0.25), Yuanyuan Feng (0.23), and Lijun Bai (0.23) (Table 8). Meanwhile, we analyzed the strongest citation bursts of the top 25 cited authors (Figure 7B). Burst detection aimed to identify an entity that was associated with a numeric function and the value of the function surges at least within a short period of time during the time frame we observed. Authors with a burst of occurrences indicate rising stars with spectacular productivity. Cristian T was the author with the strongest citation bursts, from 2014 to 2017, indicating that his articles had a great impact when it was published.

**Table 7:** Top 10 cited authors with the highest citations related to acupuncture for NDs.

**Rank**	**Citation**	**Cited author (affiliation)**
1	71	Seung-Yeon Cho (College of Korean Medicine)
2	63	Myeong Soo Lee (Universities of Exeter and Plymouth)
3	62	Jing Zhou (Beijing University of Chinese Medicine)
4	62	Wang Ying (University Hospital of Würzburg)
5	61	Bai-Yun Zeng (King's College London)
6	56	Yujie Jia (Tianjin University of Traditional Chinese Medicine)
7	53	Zhiqun Wang (Capital Medical University)
8	52	Seung-Nam Kim (Kyung Hee University)
9	52	Sujung Yeo (Kyung Hee University)
10	49	Xuying Li (The Fourth Military Medical University)

**Table 8:** Top 10 highest centrality of cited authors related to acupuncture for NDs.

**Rank**	**Centrality**	**Cited author (affiliation)**
1	0.33	Christopher G Goetz (Rush University Medical Center)
2	0.33	Jong-In Kim (Kyung Hee University)
3	0.25	Xuying Li (The Fourth Military Medical University)
4	0.23	Yuanyuan Feng (Chinese Academy of Sciences)
5	0.23	Lijun Bai (Xi'an Jiaotong University)
6	0.22	Minghua Chen (Harvard Medical School)
7	0.18	Shangjie Chen (Southern Medical University)
8	0.18	Tetsuya Asakawa (Hamamatsu University School of Medicine)
9	0.17	Seung-Tae Kim (Pusan National University)
10	0.13	Weiguo Dong (Fujian University of Traditional Chinese Medicine)

### Analysis of co-cited references

**Figure 8:**
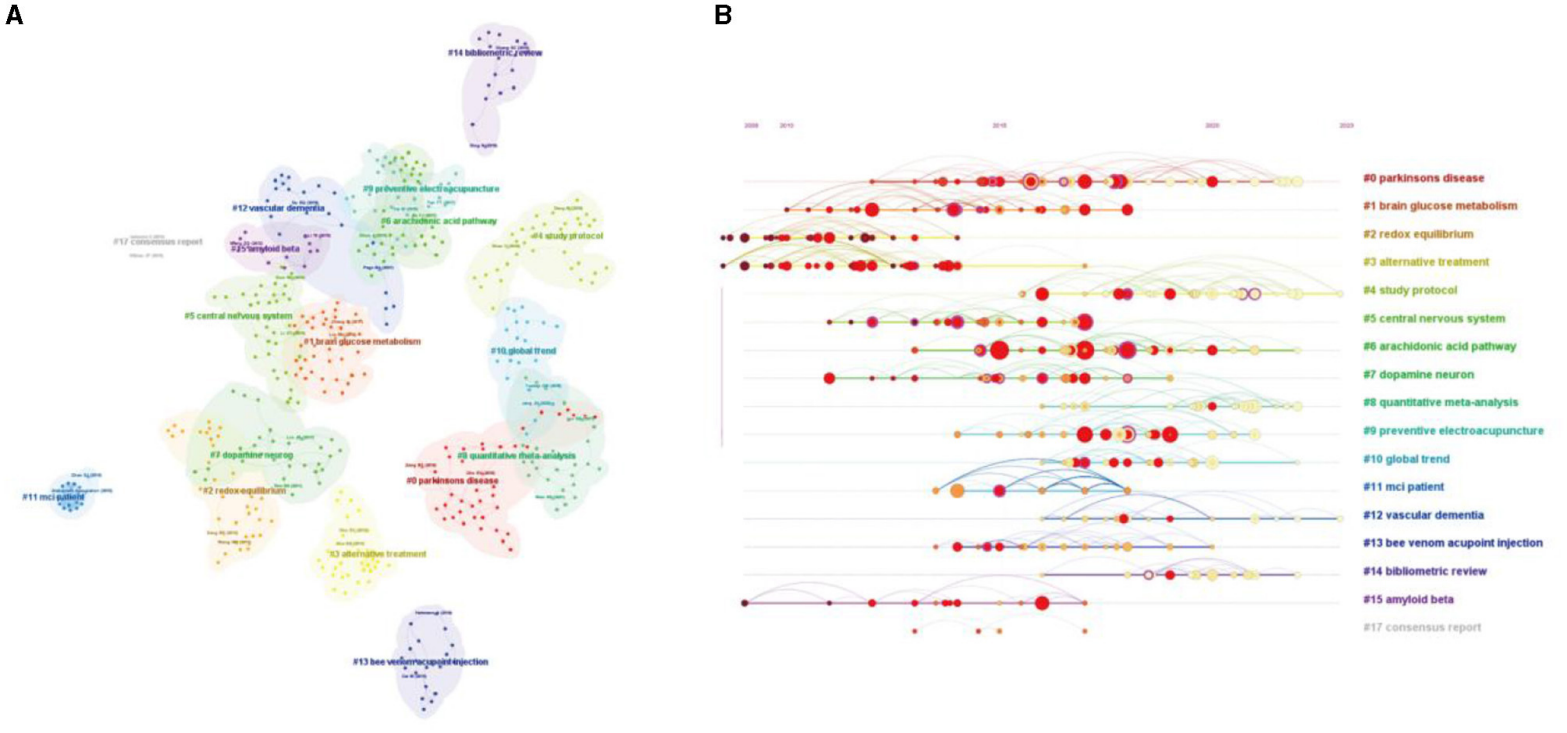
Analysis of co-citation references related to research acupuncture for NDs. **(A)** The network map of co-citation clusters. In total, 17 clusters with different research topics were formed, reflecting different colors on the map. **(B)** Timeline visualization of co-citation clusters. Each horizontal row represented a cluster, and each node on the line denoted a co-citation reference. The co-citation relationship between the two references is represented as a line connecting two nodes, and the size of the node means the number of co-cited times.

In the 635 publications retrieved, 23,436 references were cited. We obtained the reference co-citation map in the last 10 years using CiteSpace, with 533 nodes and 2,314 links. Moreover, the top 10 most popular cited references and the highest centrality cited references are listed in Tables 9, 10. We performed a cluster analysis of the co-citation references in order to uncover common themes in similar articles (Figure 8A). A total of 53 clusters were obtained. Cluster ID is the number after clustering, and the number is shown in the figure as # 0, # 1,. The larger the size of the cluster (that is, the larger the number of members included in the cluster), the smaller the number of Cluster ID. Size represented the number of members contained in the cluster. According to the log-likelihood ratio algorithm in the CiteSpace, based on the title, the five largest clusters were “Parkinson's disease (Cluster #0, size = 43, Silhouette = 0.946),” “brain glucose metabolism (Cluster #1, size = 43, Silhouette = 0.897),” “redox equilibrium (Cluster #2, size = 38, Silhouette = 0.963),” “alternative treatment (Cluster #3, size = 37, Silhouette = 0.983),” “study protocol (Cluster #4, size = 37, Silhouette = 0.958)” (Figure 8A). CiteSpace uses two metrics to assess the effectiveness of clusters, which are modularity and silhouette. According to the results, the cluster structure was significant and highly reliable, with a total modularity of 0.8565 and a weighted mean silhouette is 0.9443. Meanwhile, we further analyzed the evolution of these clusters on a timeline (Figure 8B), revealing that clusters #0, #4, and #12 were identified as the most recent regions.

**Table 9:** Top 10 most popular co-cited references related to acupuncture for NDs.

**Rank**	**Frequency**	**References**	**Author and publication year**	**Journal**
1	43	Acupuncture for patients with mild to moderate Alzheimer's disease: a randomized controlled trial (Jia et al., 2017)	Jia YJ, 2017	BMC complementary and alternative medicine
2	37	The effectiveness and safety of acupuncture for patients with Alzheimer disease: a systematic review and meta-analysis of randomized controlled trials (Zhou et al., 2015)	Zhou J, 2015	Medicine
3	31	Electroacupuncture attenuates cognition impairment via anti-neuroinflammation in an Alzheimer's disease animal model (Cai et al., 2019)	Cai M, 2019	Journal of neuroinflammation
4	23	Effectiveness of acupuncture and bee venom acupuncture in idiopathic Parkinson's disease (Cho et al., 2012)	Cho SY, 2012	Parkinsonism and related disorders
5	23	Behavioral Changes and Hippocampus Glucose Metabolism in APP/PS1 Transgenic Mice via Electro-acupuncture at Governor Vessel Acupoints (Cao et al., 2017)	Cao J, 2017	Frontiers in aging neuroscience
6	23	Effect of Acupuncture on the Motor and Nonmotor Symptoms in Parkinson's Disease–A Review of Clinical Studies (Zeng and Zhao, 2016)	Zeng BY, 2016	CNS neuroscience and therapeutics
7	23	Modulatory effects of acupuncture on brain networks in mild cognitive impairment patients (Tan et al., 2017)	Tan TT, 2017	Neural regeneration research
8	22	Manual Acupuncture Regulates Behavior and Cerebral Blood Flow in the SAMP8 Mouse Model of Alzheimer's Disease (Ding et al., 2019b)	Ding N, 2019	Frontiers in neuroscience
9	21	Electroacupuncture decreases cognitive impairment and promotes neurogenesis in the APP/PS1 transgenic mice (Li et al., 2014)	Li XY, 2014	BMC complementary and alternative medicine
10	21	Efficacy of Combined Treatment with Acupuncture and Bee Venom Acupuncture as an Adjunctive Treatment for Parkinson's Disease (Cho et al., 2018)	Cho SY, 2018	Journal of alternative and complementary medicine

**Table 10:** Top 10 highest centrality co-cited references related to acupuncture for NDs.

**Rank**	**Centrality**	**References**	**Author and publication year**	**Journal**
1	0.37	Neuronal Specificity of Acupuncture in Alzheimer's Disease and Mild Cognitive Impairment Patients: A Functional MRI Study (Shan et al., 2018)	Shan Y, 2018	Evidence-based complementary and alternative medicine
2	0.26	Electroacupuncture decreases cognitive impairment and promotes neurogenesis in the APP/PS1 transgenic mice (Li et al., 2014)	Li XY, 2014	BMC complementary and alternative medicine
3	0.25	FMRI connectivity analysis of acupuncture effects on the whole brain network in mild cognitive impairment patients (Feng et al., 2012)	Feng YY, 2012	Magnetic resonance imaging
4	0.24	Acupuncture as Adjuvant Therapy for Sleep Disorders in Parkinson's Disease (de Amorim Aroxa et al., 2017)	Aroxa FHD, 2017	Journal of acupuncture and meridian studies
5	0.23	Modulation of functional activity and connectivity by acupuncture in patients with Alzheimer disease as measured by resting-state fMRI (Zheng et al., 2018)	Zheng WM, 2018	PloS ONE
6	0.23	A study of the effects of 8-week acupuncture treatment on patients with Parkinson's disease (Yeo et al., 2018)	Yeo S, 2018	Medicine
7	0.18	Epidemiology of complementary and alternative medicine use in patients with Parkinson's disease (Wang et al., 2013)	Wang Y, 2013	Journal of clinical neuroscience
8	0.17	Electroacupuncture Suppressed Neuronal Apoptosis and Improved Cognitive Impairment in the AD Model Rats Possibly via Downregulation of Notch Signaling Pathway (Guo et al., 2015)	Guo HD, 2015	Evidence-based complementary and alternative medicine
9	0.16	A clinical study of integrating acupuncture and Western medicine in treating patients with Parkinson's disease (Chen et al., 2015)	Chen FP, 2015	American journal of Chinese medicine
10	0.16	Does integrative medicine enhance balance in aging adults? Proof of concept for the benefit of electroacupuncture therapy in Parkinson's disease (Toosizadeh et al., 2015)	Toosizadeh N, 2015	Gerontology

### Analysis of keywords

**Figure 9:**
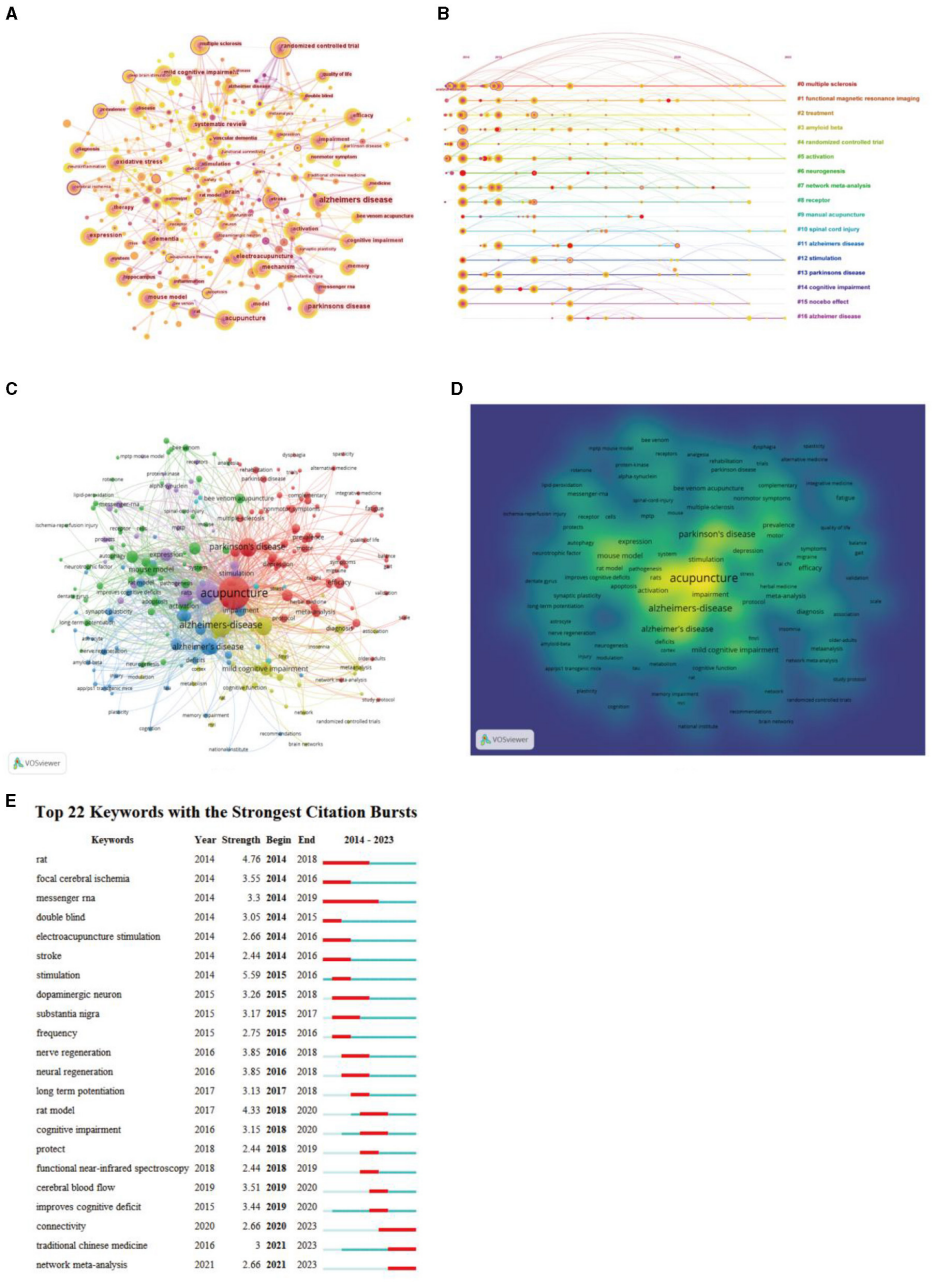
The analysis of keywords related to research acupuncture for NDs from 2014 to 2023. **(A)** Map of keywords by CiteSpace. **(B)** Timeline visualization and clustering analysis of the keywords co-occurrence network. Each horizontal row represented a cluster, and each node on the line denoted a keyword. The co-occurrence relationship between the two keywords is represented as a line connecting two nodes, and the size of the node means the number of co-occurrence times. **(C)** Network visualization of keywords used by VOSviewer. **(D)** Density visualization of keywords used by VOSviewer. **(E)** The top 22 keywords with the strongest citation bursts by CiteSpace. The beginning of a blue line represents when an article was published. The beginning of a red mark was the beginning of a period of burst, and the end of the red mark was the end of the burst period.

The analysis of keywords can add richer interpretations to understanding the research center in this field. The map of keyword co-occurrence consisted of 332 nodes and 2,374 links. The top five most frequently used keywords were “Alzheimer's disease,” “Parkinson's disease,” “acupuncture,” “dementia,” and “electroacupuncture.” The top five keywords in terms of centrality were “cerebral ischemia,” “acupuncture stimulation,” “fMRI,” “apoptosis,” and “deep brain stimulation” (Figure 9A). According to the log-likelihood ratio algorithm in the CiteSpace software, a total of 18 clusters were obtained (Figure 9B). The five largest clusters were “multiple sclerosis” (Cluster #0, size = 35, Silhouette = 0.895), “functional magnetic resonance imaging” (Cluster #1, size = 27, Silhouette = 0.872), “treatment” (Cluster 2, size = 25, Silhouette = 0.894), “amyloid beta” (Cluster #3, size = 25, Silhouette = 0.792), and “randomized controlled trial” (Cluster #4, size = 24, Silhouette = 0.928). VOSviewer was used to draw different visual clustering maps of keywords used in the published articles. The network visualization and frequency heat map of keywords were created on VOSviewer (Figures 9C, D). “Burst words” are keywords frequently used within a given period of time. It was considered that the indicators for evaluating the most cutting-edge themes or emerging trends were the increased number of keywords burst in a citation or the increased frequency of keywords within a certain period of time. As shown in Figure 9E, it highlighted the top 22 keywords with the strongest citation bursts, indicating periods of intense research activity around and interest in this field. The top five burst keywords were “stimulation,” “rat model,” “rats,” “nerve regeneration,” and “nerval regeneration.” In addition, the emergent keywords that lasted until 2023 were connectivity (2020–2023), traditional Chinese medicine (2021–2023), and network meta-analysis (2020–2023).

## Discussion

NDs may have genetic, environmental, or complex etiologies (Temple, 2023). They have become a leading cause of burden on society in recent years. A study predicted an economic burden of NDs of ~$10 trillion in 2050 globally (Nandi et al., 2022). For most NDs, there are no effective treatments (Yang et al., 2021). Although NDs are currently incurable, some treatments are available to alleviate symptoms. Currently, treatments include both pharmacological and non-pharmacological approaches. For example, cholinesterase inhibitors (donepezil, rivastigmine, and galantamine) (Li et al., 2018; Sharma, 2019; Marucci et al., 2021) and the NMDA receptor (Ahmed et al., 2020) antagonist memantine for AD, levodopa (L-DOPA) administration for PD (Teymourian et al., 2022), and transcranial magnetic stimulation (TMS) as a non-invasive non-pharmacological method for many kinds of NDs (Ni and Chen, 2015). Acupuncture was considered a non-pharmacological therapy, and its effectiveness and safety have been abundantly proven in clinical and experimental studies. In order to investigate the field of acupuncture for treating NDs, we performed a bibliometric analysis with CiteSpace and VOSviewer by searching the Web of Science Core Collection in the last decade. Based on this, our study summarized the general information and global research trends in this field.

According to the analysis of the data obtained, in the past 10 years, the overall trend of the number of annual publications on acupuncture treatment of NDs is increasing. This trend indicates that acupuncture is gradually being recognized and considered as having great potential as a complementary therapy for NDs. In terms of the publications of countries/regions, many countries and regions have paid great attention to this area. Among them, China (*n* = 389) was the most productive country, followed by South Korea, USA, Germany, and Australia. Meanwhile, half of the 10 most productive prolific and the highest centrality scientific institutions were located in China, which partially supported that China maintains a leading position regarding research into social participation in acupuncture treatment for NDs. These results were roughly consistent with previous acupuncture-related bibliometric studies (Pei et al., 2019; Huang et al., 2021), which may be attributed to the reason that acupuncture, as a part of traditional Chinese medicine, derived from China and has been practiced in China for more than 3000 years (Zhuang et al., 2013).

As for the analysis of journals and cited journals, studies on acupuncture for NDs are more popular in the Journal of Evidence-based Complementary and Alternative Medicine, which is focused on the therapy of traditional Chinese medicine. PLoS One had the highest number of citations. Some most cited references were from high-quality journals, suggesting that scholars need to expand their choices to publish the studies in high-quality journals, which helps to gain more attention about acupuncture for NDs worldwide.

From the perspective of authors and cited authors, Li Zhigang, from the Beijing University of Chinese Medicine, was the most prolific author, who had published 18 articles in this field. He mainly studied the mechanism of acupuncture on AD. For example, he found that electroacupuncture (EA) and manual acupuncture (MA) were better efficacy in the treatment of AD by improving spatial learning and memory ability via animal experiments, applied to micro-positron emission tomography (micro-PET) and arterial spin-labeling MRI for glucose metabolism and cerebral blood flow (CBF). The results showed that acupuncture could enhance glucose metabolism in the hippocampus and CBF in the prefrontal lobe (Cao et al., 2017; Ding et al., 2019b), which were consistent with the results in the co-citation references clustering results we analyzed. Recently, he further investigated the effect of MA on the intestinal mucosal barrier and the gut microbiota mechanism through which this effect occurs for AD (Hao et al., 2022). His studies revealed the mechanism and function of acupuncture for NDs, which made great contributions to the development of this field.

Reference co-citation refers to two (or more) papers being cited by one or more papers simultaneously, and it is used to assess the degree of correlation between the papers. The relationships identified through reference co-citation can vary over time, and studying these relationships allows the exploration of the development and evolutionary dynamics of a particular field of study (Huang et al., 2021). Among them, the first highest co-citation reference was a randomized controlled trial, about acupuncture for patients with mild-to-moderate AD, which mainly explored the efficacy and safety of acupuncture in patients with mild-to-moderate AD. As far as the type of co-citation reference is concerned, the vast majority of important and popular co-citation references were clinical trials and animal research studies, including AD and PD, which indicated that more attention has been paid to the clinical efficacy of acupuncture and its mechanism research for NDs. However, there is a lack of review to summarize them. In a cluster analysis of the co-citation references, based on title, knowledge on “Parkinson's disease,” “brain glucose metabolism,” “redox equilibrium,” “alternative treatment,” and “study protocol” were the five largest groups of research. Among them, although PD is the second most common neurodegenerative disorder after AD (de Lau and Breteler, 2006), there were many related studies on acupuncture intervention in PD, including clinical trials (Jang et al., 2020a; Fan et al., 2022) and mechanism research involving animals (Jang et al., 2020b; Oh et al., 2022). Some research studies have demonstrated NDs may have a glucose metabolism disorder in the brain. According to the image of PET-CT, acupuncture could activate brain glucose metabolism to ameliorate cognitive impairment (Yang et al., 2014; Liu et al., 2017). These studies and results further prove that acupuncture may improve the symptoms of NDs by regulating brain glucose metabolism, which has also been the research focus in recent years. According to cluster analysis of the co-citation references, it can be observed that researchers use different technical means to study the mechanism of acupuncture in treating NDs. Acupuncture has also been recognized as an alternative treatment for NDs.

The top five most frequently used keywords were “Alzheimer's disease,” “Parkinson's disease,” “acupuncture,” “dementia,” and “electroacupuncture.” AD and PD are the most common diseases of NDs. Therefore, a large amount of research on NDs is also based on these two disease types, and the clinical manifestations of these diseases also include dementia. However, the top five keywords in terms of centrality were “cerebral ischemia,” “acupuncture stimulation,” “fMRI,” “apoptosis,” and “deep brain stimulation.” Cerebral ischemia is usually associated with stroke, while Anja Kahl et al. found that cerebral ischemia induces the aggregation of proteins linked to NDs, which reveals a previously unappreciated molecular overlap between neurodegenerative diseases and ischemic stroke (Kahl et al., 2018). Interestingly, the keyword “cerebral blood flow” was one of the top 22 keywords with the strongest citation bursts. It indicates that previous studies have focused on the effect of acupuncture on cerebral blood flow to alleviate the symptoms of NDs (Ding et al., 2019a,b; Sun et al., 2019).

A growing number of evidence increasingly validates the practice of acupuncture for NDs. The study from this bibliometric study provides insight into the research trends in acupuncture therapy for NDs, and the current status and trends of the past decade, which may help researchers confirm the current status, hotspots, and frontier trends in this field. The mechanism research studies in this field are gradually deepening, with the development of science and technology, especially the application of PET, fMRI, and ultrasound in recent years. Combining molecular biology experiments, these articles indicated that acupuncture treatment of NDs is closely related to the mechanism of regulating redox equilibrium, reducing neurotoxic Aβ, alleviating cytokine-induced inflammatory response, and improving cerebral blood perfusion. Although the mechanism of acupuncture treatment for NDs is being studied in depth, there is still a lot of room for exploration based on the currently published articles. For example, whether it is possible to combine omics technology and single-cell sequencing technology to study this field in the future, in order to achieve greater breakthroughs. Based on the published articles of the past decade, there are relatively few high-quality clinical studies. Through the results of our analysis, we need high-quality clinical trials to further supplement its scientific nature in the future. Meanwhile, it is important to strengthen the cooperation between different countries and different institutions, which is conducive to further promoting the research development of acupuncture for the treatment of NDs.

## Limitations

There are some limitations to this study. First of all, we only analyzed publications of the Web of Science database, and the language is English. It was possible that some studies related to this area have not been included. Second, this study included both articles and reviews and the uneven quality of the collected studies may reduce the credibility of the map analysis. Finally, the focus of this study was that NDs do not include frontotemporal dementia (FTD) and dementia with Lewy bodies (DLB), because there was limited clinical application of acupuncture for these two diseases, and research on acupuncture for these two diseases was almost impossible to search on WOS. The reasons behind this are also worth digging into and exploring in the future. However, the visualized analysis based on literature studies undoubtedly lays a foundation for scholars to quickly understand the research subjects, research hotspots, and development trends in the field of acupuncture for NDs.

## Author contributions

QT: Writing—original draft, Writing—review & editing, Conceptualization. XL: Data curation, Writing—review & editing. SX: Writing—review & editing. JC: Data curation, Writing—review & editing. WL: Data curation, Writing—review & editing. SZ: Writing—review & editing. YD: Funding acquisition, Writing—review & editing, Conceptualization.
